# A Case of Type 1 Diabetes Mellitus With Intractable Chronic Diarrhea and Challenging Glycemic Control

**DOI:** 10.7759/cureus.72070

**Published:** 2024-10-21

**Authors:** Shoko M Yamada, Hitomi Takada, Takane Harada, Shozo Terada, Yoshio Nehashi, Noriko Mori

**Affiliations:** 1 Neurosurgery, Teikyo University Hospital, Mizonokuchi, Kawasaki, JPN; 2 Internal Medicine, JCHO Sakuragaoka Hospital, Shizuoka, JPN

**Keywords:** antidiarrheal agents, diabetes mellitus, diarrhea, glycemic control, steroids

## Abstract

The first step in treating diabetes mellitus (DM)-related diarrhea is to thoroughly control the serum glucose level. The authors herein describe a 75-year-old man who was diagnosed with type 1 DM when he was 32 years old. The patient lost his appetite due to hot weather and spent a week eating sweets and drinking juice. As a result, he developed diabetic ketoacidosis and was initially treated in another hospital, and then transferred to our facility for glycemic control two weeks later. He had been suffering from watery diarrhea for more than 100 days after being admitted to our hospital. His blood glucose control was extremely difficult, fluctuating between 30 and 500 mg/dL while watery diarrhea continued, and microscopic colitis was diagnosed by histological examination through endoscopy. Despite the use of insulin, multiple antidiarrheal agents including salazosulfapyridine, and a switch to total parenteral nutrition, his blood glucose fluctuations did not improve. In addition, his liver function deteriorated, his renal function declined, and hypoproteinemia developed. Considering drug-induced liver dysfunction, steroids were administered despite the high risk of further poor glycemic control. Following steroid administration, his liver function rapidly improved and the watery diarrhea resolved within a few days. Contrary to our concerns, his blood glucose level stabilized between 100 and 200 mg/dL. The mechanism underlying the stabilization of blood glucose levels after steroid use remains unclear. Prior to steroid administration, the frequency of diarrhea had been reduced from more than five times a day to only once or twice a day with antidiarrheal agents; nevertheless, the patient’s glycemic control worsened. Therefore, the stabilization of his blood glucose cannot simply be attributed to the resolution of the diarrhea. This case suggests that steroid administration may be a consideration in patients with DM-related watery diarrhea who show a poor response to antidiarrheal agents and severe blood glucose fluctuations.

## Introduction

Chronic diarrhea is defined as the passage of loose or liquid stools with increased frequency (more than three times per day) or an output exceeding 200 g per day for more than four weeks [1]. The diarrhea is observed in 8-22% of diabetic patients and often leads to impaired quality of life [2,3], and it occurs more frequently in those with type 1 diabetes mellitus (DM) than in those with type 2 DM [4]. Medications for DM, such as metformin, acarbose, and miglitol, can cause diarrhea [3], but the term “diabetic diarrhea” was first introduced by Bargen et al. in 1936 to describe unexplained diarrhea associated with DM [5]. The pathogenesis of diabetic diarrhea has been reported to include anorectal dysfunction, abnormal intestinal motility and secretion, bacterial overgrowth in the small intestine, bile acid deficiency, pancreatic exocrine insufficiency, and celiac disease; however, they have not been clearly identified [6]. It has been reported that most cases presenting with diabetic diarrhea have poor glycemic control and exhibit symptoms of autonomic neuropathy [7, 8]. The primary treatment for diabetic diarrhea is to improve glycemic control, followed by discontinuation of diarrhea-inducing drugs; adherence to a diet low in fermentable oligosaccharides, disaccharides, monosaccharides, and polyols (commonly known as FODMAPs); and the use of antidiarrheal agents [8,9]. Microscopic colitis, an autoimmune-mediated disease, was recently reported to be significantly associated with type 1 DM based on statistical evidence, although the underlying mechanism remains unclear [10,11]. We herein describe a patient with type 1 DM who developed DM-related watery diarrhea, microscopic colitis, and poor glycemic control. In this case, good glycemic control could not be achieved without amelioration of the diarrhea.

## Case presentation

**Figure 1:**
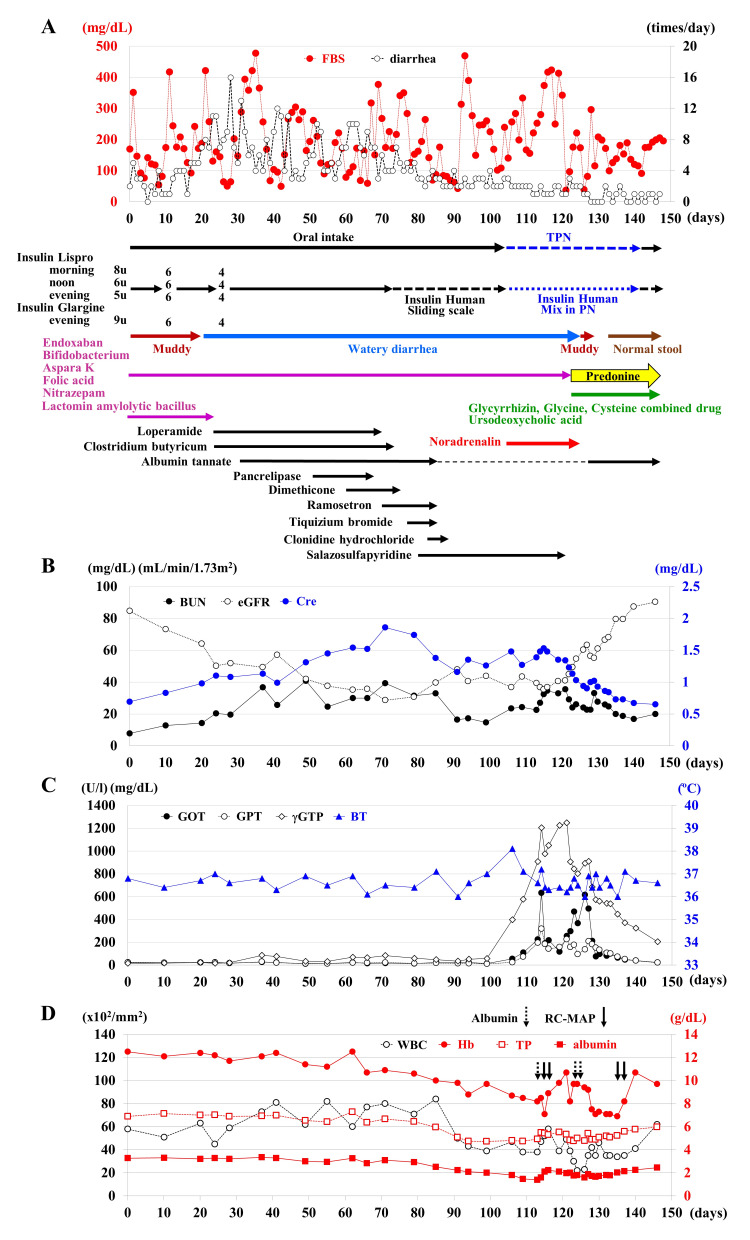
Chronological changes in medication and laboratory data (A) Watery diarrhea has appeared since day 20 despite the administration of antidiarrheal agents. From days 40 to 70, the fasting blood sugar (FBS) levels were controlled between 60 and 300 mg/dL despite the occurrence of more than four episodes of diarrhea per day. After day 70, the frequency of daily diarrhea decreased, but FBS continued to fluctuate between 50 and 450 mg/dL. Total parenteral nutrition (TPN) mixed with a fixed dose of insulin was initiated from day 104, but the blood glucose fluctuations did not improve. Following the administration of prednisolone on day 122, the FBS levels stabilized between 100 and 200 mg/dL, and stools change to muddy from watery within a few days. (B) The blood urea nitrogen (BUN) was maintained below 40 mg/dL with supplemental fluid transfusions. The creatinine level consistently exceeded 1.0 mg/dL after day 40, and the estimated glomerular filtration rate (eGFR) fluctuated around 40 mL/min/1.73 m^2^. After the administration of prednisolone, the creatinine and eGFR values rapidly returned to the reference ranges. (C) After administration of salazosulfapyridine, the r-glutamyl transpeptidase (rGTP) level began to increase followed by rise in the glutamic oxaloacetic transaminase (GOT) and glutamic pyruvic transaminase (GPT) values. When the rGTP level exceeded 1200 U/L, the GOT and GPT levels exceeded 600 U/L and 300 U/L, respectively, prednisolone administration was initiated, leading to dramatic improvement of liver function. (D) The white blood cell (WBC) count, hemoglobin, total protein (TP), and albumin values began to decrease when administration of salazosulfapyridine was initiated. During the period of abnormally elevated liver enzymes, the WBC count, hemoglobin, TP, and albumin reached their lowest levels. The WBC count dropped to 2000/mm^3^ and recovered after administration of prednisolone. Transfusion of red blood cells and administration of albumin products were required to manage the anemia and hypoalbuminemia.

A 75-year-old man lost his appetite due to hot weather and spent a week eating sweets and drinking juice. As a result, he developed diabetic ketoacidosis, was hospitalized in another facility for treatment, and was transferred to our hospital two weeks later for glycemic control. Type 1 DM was diagnosed when he was 32 years old, based on the data that glutamic acid decarboxylase (GAD) antibody was positive and C-peptide was completely depleted requiring insulin therapy at the time of the DM diagnosis. He used to drink 500 ml of beer and smoke 20 cigarettes a day but has neither drunk nor smoked since age 32 when he was diagnosed with diabetes. Upon admission to our hospital, he was able to walk with a cane and independently consume soft food. His daily caloric intake was restricted to 1400 kcal and he was on a regular insulin regimen, receiving insulin lispro before each meal and insulin glargine before the evening meal (Fig. 1A).

**Figure 2:**
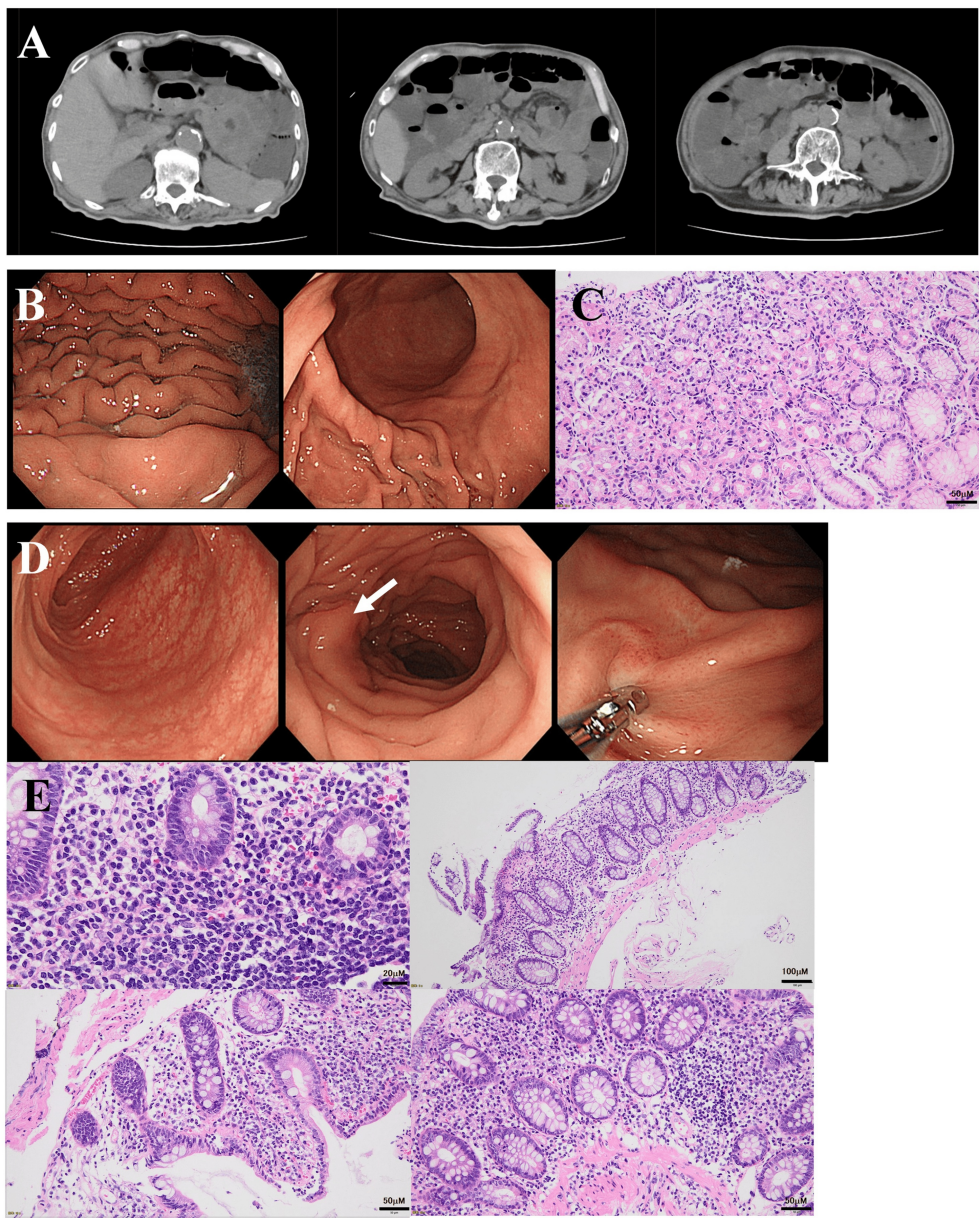
Diagnosis of chronic microscopic colitis (A) Abdominal computed tomography scan on day 51 revealed gas and water retention without solid stools in the intestinal tract. (B) Gastroscopy on day 52 showed that the gastric mucosa was completely intact. (C) No histological abnormalities were identified in the gastric mucosa. (D) Colonoscopy on day 57 demonstrated no inflammation in the colonic mucosa, but mildly thickened folds (white arrow) were intermittently present from the ileum to the sigmoid colon. (E) Histological investigations showed increased submucosal neutrophils, eosinophils, and lymphocytes in the ileum (upper left), transverse colon (upper right), descending colon (lower left), and sigmoid colon (lower right).

The patient had been experiencing loose stools since the onset of diabetic ketoacidosis and had been passing muddy stools two to three times daily. His oral medications, including edoxaban tosylate hydrate, *Bifidobacterium*, potassium L-aspartate, folic acid, nitrazepam, and lactomin/amylolytic bacillus, were continued after admission. Following admission, his fasting blood glucose levels fluctuated between 50 and 420 mg/dL (Fig. 1A). From day 20 of admission, he began passing non-bloody watery stools daily (Fig. 1A) despite the fact that his thyroid hormone levels were within the reference range, *Clostridium difficile *toxin was negative, and *Escherichia coli *1+ was the only organism identified in stool cultures. The lactomin/amylolytic bacillus was discontinued, and loperamide and *Clostridium butyricum* MIYAIRI were newly prescribed. However, the watery stools persisted, occurring more than 10 times per day even after the addition of albumin tannate on day 30. The patient never complained of abdominal pain at all despite continuing watery diarrhea, and neither abdominal distention nor tenderness was found. An abdominal computed tomography (CT) scan on day 51 revealed water retention in the intestines without organic abnormalities (Fig. 2A). Gastroscopy on day 52 showed no abnormal findings, either grossly or histologically, in the mucosa from the esophagus to the stomach (Fig. 2B, 2C). Colonoscopy on day 57 showed no ulcers or inflammation in the colonic mucosa, although mildly thickened areas were observed intermittently from the ileum to the sigmoid colon (Fig. 2D). Histological examination revealed an increase in lymphocytes, neutrophils, and eosinophils in the submucosa from the ileum to the sigmoid colon, suggesting mild chronic colitis (Fig. 2E).

Despite trials with pancrelipase, dimethicone, ramosetron, tiquizium bromide, clonidine hydrochloride, and salazosulfapyridine, the frequency of diarrhea decreased but the stools remained watery. The blood glucose level continued to fluctuate (Fig. 1A), and renal function gradually deteriorated as indicated by an increasing creatinine level and decreasing estimated glomerular filtration rate despite intravenous fluid supplementation (Fig. 1B). The liver enzymes began to rise from day 100 (Fig. 1C), and by day 104, the patient had become somnolent, developed a fever of higher than 38°C, and experienced a drop in blood pressure to 72/48 mmHg, necessitating a norepinephrine infusion and the initiation of total parenteral nutrition.

**Figure 3:**
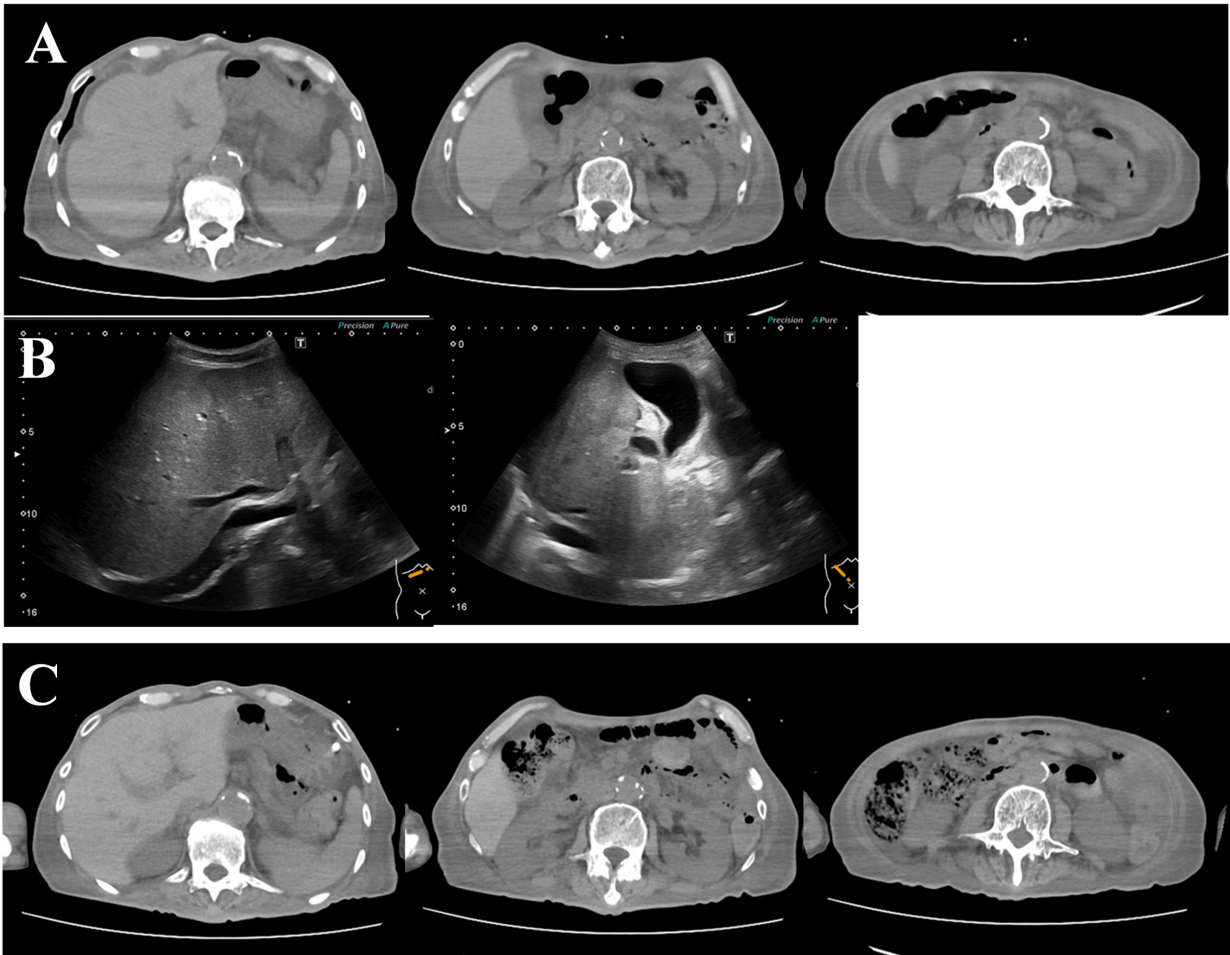
Before and after administration of prednisolone (A) On day 115, computed tomography revealed fluid accumulation in the bowel, ascites, and subcutaneous edema over the trunk due to hypoproteinemia. (B) Abdominal ultrasonography showed no inflammation in the liver or gall bladder. (C) Abdominal computed tomography on day 142 showed the accumulation of solid stool in the ascending and transverse colon and a decrease in the ascites.

On day 115, the γ-glutamyl transpeptidase level exceeded 1200 U/L, the glutamic oxaloacetic transaminase level exceeded 600 U/L, and the glutamic pyruvic transaminase level exceeded 300 U/L (Fig. 1C). However, the ammonia and total bilirubin levels remained within the reference range. Transfusion of concentrated red blood cells and administration of albumin products were required because of the progression of anemia and hypoproteinemia (Fig. 1D). An abdominal CT scan on day 115 showed ascites, fluid accumulation in the bowel, and subcutaneous edema over the trunk (Fig. 3A); however, no inflammation was observed in the liver or gallbladder on abdominal sonography (Fig. 3B). The white blood cell count had gradually declined since the initiation of salazosulfapyridine (Fig. 1D); therefore, considering potential drug side effects, the salazosulfapyridine was withdrawn and intravenous administration of a hepatoprotective drug and 30 mg of prednisolone was started on day 122.

By day 126, the watery diarrhea had changed to muddy stools, and after 5 days of constipation, normal stool was observed on day 132. The diarrhea did not recur thereafter (Fig. 1A). The urinary glucose level, which had been 948 mg/dL before steroid administration, decreased to ≤20 mg/dL, and the blood glucose level stabilized at 100 to 200 mg/dL after steroid administration (Fig. 1A). An abdominal CT scan on day 142 demonstrated a reduction in ascites and accumulation of solid stool in the ascending and transverse colon (Fig. 3C). The patient recovered rapidly, regained the ability to speak, and resumed normal oral intake, although he developed significant disuse syndrome. He was transferred to a rehabilitation hospital on day 147, with a maintenance dose of 10 mg of oral prednisolone.

## Discussion

Distinguishing diabetic diarrhea from non-diabetic diarrhea can be challenging. In our case, diarrhea began as muddy stools during a period of poor glycemic control following diabetic ketoacidosis and progressed to watery diarrhea with prolonged poor glycemic control. This suggests a potential relationship between poor glycemic control and the onset of diarrhea. I, the patient was diagnosed with microscopic colitis based on the long-standing non-bloody watery diarrhea and histological examinations [12,13], although whether the microscopic colitis was directly related to the patient’s type 1 DM remains unclear.

Improving glycemic control is typically the most important treatment for diabetic diarrhea [7,8]. However, in this case, achieving stable glycemic control was extremely difficult despite adjustments to the insulin regimen. Common sense would suggest that administering steroids in such a situation would complicate glycemic control, potentially requiring increased insulin doses. However, the patient developed severe drug-induced hepatitis, compounded by hypoproteinemia and declining renal function, making the treatment of hepatitis a priority over serum glucose control. Consequently, steroids were administered despite the risk of worsening glycemic control.

Budesonide is widely accepted as the first-line therapy for microscopic colitis [11]. However, Taketomi et al. used prednisolone in a diabetic patient for chronic diarrhea caused by drug-induced collagenous colitis, and the diarrhea rapidly improved [14]. Prednisolone was chosen in this case because of the severity of the drug-induced hepatitis. Following steroid administration, the patient’s liver function rapidly improved, the diarrhea that had persisted for 100 days was completely resolved within a few days, and the blood glucose fluctuations stabilized between 100 and 200 mg/dL. Although the anti-inflammatory effects of steroids likely cured both the drug-induced hepatitis and chronic enteritis, the stabilization of the blood glucose level following steroid administration is more difficult to explain.

Given that this patient had type 1 DM and thus a severely limited capacity for insulin secretion, it is unlikely that the blood glucose fluctuations were due to unstable insulin production. Instead, the fluctuations may have been related to irregular nutrient absorption from the gut secondary to chronic colitis. However, this does not fully explain why the fluctuations persisted even after switching to total parenteral nutrition.

In general, the first step in treating DM-related watery diarrhea is to achieve good glycemic control [7,8]. However, this case highlights that in some instances, good glycemic control may not be achievable until the watery diarrhea is fully resolved. In our case, even after day 90, when the frequency of diarrhea decreased, the blood glucose level remained highly variable.

## Conclusions

In chronic diarrhea associated with DM, it is important to discontinue drugs that may cause diarrhea, to stabilize blood glucose levels, and to use proper antidiarrheal agents.

However, when the blood glucose level dramatically fluctuates from hypoglycemia to hyperglycemia under insulin administration rather than remaining continuously hyperglycemic, steroid therapy should be considered to stop diarrhea. Although the possibility of worsening glycemic control with steroid administration cannot be ruled out, there is a possibility that blood glucose levels may settle down.
